# Blood eosinophil count predicts treatment failure and hospital readmission for COPD

**DOI:** 10.1183/23120541.00188-2020

**Published:** 2020-11-10

**Authors:** Marjan Kerkhof, Isha Chaudhry, Ian D. Pavord, Marc Miravitlles, Chin Kook Rhee, David M.G. Halpin, Omar S. Usmani, Rupert Jones, Janwillem Kocks, Marianna Alacqua, Tamsin Morris, Alan Kaplan, David B. Price

**Affiliations:** 1 Observational and Pragmatic Research Institute, Singapore, Singapore; 2 Oxford Respiratory NIHR BRC, Nuffield Dept of Medicine, University of Oxford, Oxford, UK; 3 Pneumology Dept, Hospital Universitari Vall d'Hebron, Vall d'Hebron Institut de Recerca (VHIR), Vall d'Hebron Barcelona Hospital Campus, CIBER de Enfermedades Respiratorias (CIBERES), Barcelona, Spain; 4 College of Medicine, Seoul St Mary's Hospital, The Catholic University of Korea, Seoul, South Korea; 5 University of Exeter Medical School, College of Medicine and Health, University of Exeter, Exeter, UK; 6 Imperial College London, London, UK; 7 The Peninsula College of Medicine and Dentistry, Plymouth, UK; 8 AstraZeneca, Cambridge, UK; 9 AstraZeneca, Luton, UK; 10 Family Physician Airways Group of Canada, Richmond Hill, ON, Canada; 11 University of Aberdeen, Aberdeen, UK

## Abstract

We examined associations between blood eosinophil counts (BEC) and risk of treatment failure or hospital readmission following acute oral corticosteroid (OCS)-treated COPD exacerbations.

We conducted studies from the Optimum Patient Care Research Database (OPCRD) (www.optimumpatientcare.org/opcrd) and Clinical Practice Research Datalink (CPRD) (www.cprd.com/home/), validated databases for medical research, with linked Hospital Episode Statistics (HES) data for ∼20 000 COPD patients aged ≥40 years. For patients with OCS-treated COPD exacerbations treated in primary care, with BECs recorded on first day of OCS treatment (Cohort 1), we assessed treatment failure (COPD-related hospitalisations and OCS prescriptions beyond index OCS course). For patients hospitalised for COPD exacerbations, with BEC measured over an exacerbation-free period during the year prior to admission (Cohort 2), we assessed readmission rate. Cox proportional hazards regression analysis was adjusted for confounders to estimate the association between BEC and treatment outcomes.

Of patients treated with OCS for COPD exacerbations in primary care (Cohort 1), 44% experienced treatment failure following single OCS courses, and 10% (255/2482) were hospitalised for ≤6 weeks. Greater BEC was associated with reduced hospital-admission risk (hazard ratio [HR]=0.26; 95% CI: 0.12–0.56, per 100 cells·µL^−1^ increase). BEC increases of ≥200 cells·µL^−1^ from exacerbation-free periods to exacerbations were associated with least hospitalisation risk (HR=0.32; 95% CI: 0.15–0.71) *versus* no BEC change. For patients hospitalised for COPD exacerbations (Cohort 2), 4-week hospital readmission was 12% (1189/10 245). BEC increases during an exacerbation-free period within the past year were associated with reduced risk of short-term readmission (HR=0.78; 95% CI: 0.63–0.96).

Greater BEC predicted better outcomes for patients with OCS-treated COPD exacerbations, whether community or hospital managed. Eosinopenia predicted worse outcomes.

## Abstract 2

**Patients who have greater blood eosinophil counts during #AECOPD in the last year have lesser hospitalisation risk, enhanced OCS response and better response to OCS upon hospitalisation, as reflected by reduced readmissions** https://bit.ly/2ZQIV1n

## Introduction

COPD is a prevalent, complex and heterogeneous disease with limited treatment success for some patients [1]. Many patients with COPD experience exacerbations, which contribute to disease progression, especially if recovery from exacerbations is slow [2, 3]. COPD exacerbations can be heterogeneous in aetiology and underlying inflammation [2]. Systemic corticosteroids are recommended for the treatment of COPD exacerbations because they can shorten recovery time and hospitalisation duration and increase time to a subsequent exacerbation [3, 4]. However, systemic corticosteroids are associated with known adverse events. Treatment strategies targeted at the needs of individual patients may improve treatment-related clinical outcomes [1].

COPD is commonly characterised by neutrophilic inflammation, but ∼25‒40% of patients with COPD have elevated blood eosinophil counts (BEC) [2, 5–7]. This may represent a different phenotype from neutrophilic COPD [8, 9]. Patients with COPD who have greater BEC have an enhanced response to corticosteroids [7, 10]. BEC have recently been recommended by the Global Initiative for Chronic Obstructive Lung Disease (GOLD) for predicting the effectiveness of inhaled corticosteroids (ICS) in preventing COPD exacerbations [3]. BEC may therefore be useful biomarkers to guide treatment decisions [3, 10, 11].

Clinical trial data suggest an increased BEC is associated with an increased likelihood of ICS treatment benefit for patients with COPD [12, 13]. An analysis of three randomised controlled trials for the treatment of acute COPD exacerbations reported reduced treatment failure with prednisolone for patients with elevated blood eosinophils (≥2% of total white blood cells) but not for those with blood eosinophils <2% [10, 11]. A recent analysis of the Phase III IMPACT trial demonstrated that regimens containing ICS reduced rates of moderate and severe exacerbations to a greater extent for patients with increasing BEC [14]. Results from previous studies are inconsistent on whether BEC at the time of hospitalisation predict readmission risk after discharge for patients with COPD [7, 15‒18]. Some studies found that patients with elevated BEC at admission had a greater risk of hospital readmission or treatment failure, but these patients may have had lesser risk of death [15, 16, 18]. Other studies reported shorter hospitalisations or reduced readmission rates for patients with elevated BEC [7, 17]. This is consistent with the known heterogeneity of COPD [2].

Although OCS are relatively inexpensive, their use in the treatment of patients hospitalised for COPD exacerbations is associated with significant short- and long-term adverse effects that increase healthcare resource costs [19]. Little is known about exacerbation treatment failure rates for patients who have received short-term OCS courses from their treating physicians or whether BEC can predict risk of short-term hospital readmission following COPD exacerbations. We performed two separate analyses of a large, real-world data population of ∼20 000 patients from validated, high-quality databases to determine better whether BEC constitute useful biomarkers of treatment response for patients with COPD. We sought to determine fully whether there was a BEC–OCS response relationship and to provide more accurate information on optimal cut-off points. Additionally, we sought to acquire more detailed information on small populations at risk (*e.g.*, group with eosinopenia (<50 cells·µL^−1^)), which can be identified at routine full blood count measurements in general practice. The first analysis examined the effect of BEC on treatment outcomes for patients treated with OCS for COPD exacerbations by general practitioners in the United Kingdom. The second analysis focused on patients who had been hospitalised because of COPD exacerbations and the relationship between BEC and risk of readmission to the hospital.

## Methods

This analysis includes two historical cohorts that were derived from COPD electronic medical records in the United Kingdom: Blood Eosinophil Counts in Guiding Anti-inflammatory Treatment of COPD Exacerbations (BLANCA; Cohort 1) and Blood Eosinophil Counts and Risk of Short-Term Hospital Readmission for COPD Exacerbation (Cohort 2). Study protocols for each cohort were approved by the Clinical Practice Research Datalink (CPRD) Independent Scientific Advisory Committee (ISAC approval numbers 16_299 and 16_297) and the Anonymised Data Ethics & Protocol Transparency Committee (ADEPT) Committee (ADEPT0917) for use of the Optimum Patient Care Research Database (OPCRD). These two studies were registered with the European Union Electronic Register of Post-Authorisation Studies (EU PAS Register numbers EUPAS17543 and EUPAS16875). Studies were performed in accordance with ethical principles consistent with the Declaration of Helsinki, International Conference on Harmonisation Good Clinical Practice and applicable legislation on non-interventional studies.

### Data sources

Primary and secondary medical care data for Cohorts 1 and 2 were obtained from two sources: 1) OPCRD (www.optimumpatientcare.org/opcrd), and 2) CPRD, a validated database frequently used for medical and health research (www.cprd.com/home/) [20] with linked Hospital Episode Statistics (HES) data. Both databases contain anonymised longitudinal electronic medical records extracted from subscribing UK primary-care practices. At the time of these analyses, OPCRD contained records for 6 million UK patients from >600 practices, and CPRD contained medical records for 5 million UK patients from >600 practices. Data from both sources, comprising ∼10 000 eligible patients, were combined to maximise statistical power. Duplicate records were removed, which yielded a combined data set of unique patients with COPD available for all analyses in both cohorts.

To obtain detailed information about hospital admissions, we identified CPRD data with linked HES admitted patient care data, which contain details of all admissions to or visits with National Health Service healthcare providers in the United Kingdom [21]. Diagnostic data of admissions recorded in HES are coded *via* the International Classification of Diseases, 10th revision (ICD-10) coding frame. To identify accident and emergency attendances, CPRD data were linked with HES accident and emergency data.

### Study designs

In both cohorts, patients were required to have been ≥40 years of age, have had continuous medical data available for ≥1 year before their index dates and have had a COPD diagnosis before their index dates. Patients were excluded if they had any chronic lower respiratory conditions other than asthma or COPD or were receiving maintenance OCS treatment (≥5 prescriptions of prednisolone ≤10 mg·day^−1^) in the year prior to the index date.

#### Patients treated with oral corticosteroids for COPD exacerbations in a primary-care setting (Cohort 1)

For Cohort 1, patients were required to have had a COPD exacerbation that occurred during or after 2005 and ≥1 exacerbation treated in primary care with OCS. Index date was the day of a prescription for OCS for COPD exacerbation, with BEC measured on the same day. Patients were required to have a COPD diagnosis within the Quality and Outcome Framework, which requires a confirmed post-bronchodilator ratio of forced expiratory volume in 1 s (FEV_1_)/forced vital capacity <0.70. Patients were not included if they had received OCS or antibiotic treatment during the 2 weeks before the exacerbation.

The primary outcome for patients treated with OCS for COPD exacerbations in a primary-care setting (Cohort 1) was overall treatment failure within 6 weeks of exacerbation. Treatment failure was defined as a COPD-related hospital admission or accident and emergency department visit, prescription for antibiotics with lower respiratory consultation or repeat OCS prescription. If patients in Cohort 1 had >1 event that met criteria for an index date, the latest event was selected.

#### Patients hospitalised due to COPD exacerbations (Cohort 2)

Patients in Cohort 2 had been admitted to a hospital in the United Kingdom for treatment of a COPD exacerbation (ICD-10 code J44.0 or J44.1 in any diagnostic position) and had BEC measured within the prior year during an exacerbation-free period. An exacerbation-free period was defined as a ≥4-week period in the year before the index date and prior to the BEC measurement during which the patient experienced no COPD exacerbations. No requirements were specified for year of hospitalisation for Cohort 2. The primary outcome was time to first hospital readmission for COPD within 4 weeks after discharge date. If patients in the group hospitalised for COPD exacerbation (Cohort 2) had >1 event that met criteria for an index date, the earliest event was selected.

### Statistical analyses

For both cohorts, a total of 10 079 patients were required to achieve an HR of 1.30 (or 0.77) with an assumption of significance at α=0.05, assuming that 10% of patients would have high BEC (≥450 cells·µL^−1^) and that risk of hospital admission or readmission during the outcome period was 10% for patients without high BEC. The risk of hospital admission or readmission was based on a cohort study by Hunter *et al*. [22], who found that 10% of readmissions occurred within 14 days of previous discharge.

Statistical analyses were conducted *via* Stata version 14 (StataCorp, College Station, TX, USA) and SPSS Statistics version 23 (IBM, Armonk, NY, USA). For both cohorts, demographics, baseline patient characteristics, comorbidities and disease severity were described. HRs with 95% confidence intervals for the association between outcome and incremental BEC categories were estimated by Cox proportional hazards regression model, with BEC 50–<150 cells·µL^−1^ as the reference category. Primary analyses compared eight eosinophil count categories with a reference category (50–<150 cells·µL^−1^). Analysing BEC as a continuous variable resulted in a slightly improved model fit (lesser Akaike information criterion/Bayesian information criterion) for hospitalisation risk as the outcome. These data are also reported.

We also examined patient characteristics, exacerbation frequency, modified Medical Research Council (mMRC) dyspnoea scale score and percentage predicted FEV_1_ as independent predictors of hospital admission (Cohort 1) and readmission (Cohort 2). For patients with several assessments of the mMRC dyspnoea scale score and FEV_1_, the most recent assessment within the past 5 years was used. Patients were censored from the analyses at the time of death. Sex, age, body mass index (BMI), smoking status, Charlson comorbidity index, comorbidities and timing of BEC relative to index date were evaluated as confounders for the effect estimate. A forward-selection procedure was employed to derive the final models. Logistic regression analysis for incremental BEC categories evaluated the relationship between BEC and treatment response. The validity of the proportional hazards assumption was checked with survival curves.

Sensitivity analyses were stratified by ICS treatment in the year before index date. Differences between strata were tested by including an interaction term in the model analysing the full population. HRs were estimated for BEC <50 cells·µL^−1^ and ≥150 cells·µL^−1^ compared with the reference, 50–<150 cells·µL^−1^.

## Results

### Patients treated with oral corticosteroids for COPD exacerbations in a primary-care setting (Cohort 1)

Index years for patients treated in a primary-care setting (Cohort 1) ranged from 2005 to 2017. We identified 8848 patients with COPD and BEC recorded on the day of an exacerbation treated by their general practitioners (see supplementary figure S1 for patient flow diagram). Of these patients, 6416 were treated with OCS for their most recent exacerbations and comprised the analysis population of Cohort 1. Most patients were aged from 60 to <80 years (table 1); 3917 (61.1%) received antibiotic treatment along with OCS at index date, and 2482 (38.7%) had records in CPRD with HES linkage. A total of 4581 patients (71.4%) had BEC measurements during an exacerbation-free period in the prior year, including 1679 records with HES linkage. Of 6416 patients treated with OCS for COPD exacerbations in a primary-care setting (Cohort 1), 88 (1.4%) died within 6 weeks of the index exacerbation. Additional baseline characteristics are reported in supplementary table S1.

**TABLE 1:** Demographics and baseline patient characteristics for patients treated with oral corticosteroids for COPD exacerbations in a primary-care setting (Cohort 1) and in a hospital setting (Cohort 2)

**Variable**	**Cohort 1**	**Cohort 2**
**Subjects**	6416	10 245
**Age years**	71.3±10.3	75.1±9.9
≥40–<60 years	850 (13.2)	716 (7.0)
≥60–<80 years	4113 (64.1)	5838 (57.0)
≥80 years	1453 (22.6)	3691 (36.0)
**Male**	3011 (46.9)	5258 (51.3)
**Smoking status^#^**		
Nonmissing	6364 (99.2)	10,169 (99.3)
Never-smoker	747 (11.7)	993 (9.8)
Current smoker	2044 (32.1)	2940 (28.9)
Ex-smoker	3573 (56.1)	6236 (61.3)
**BMI^#^** **kg·m^−2^**	27.6±6.1	26.0±6.2
Nonmissing	1769 (27.6)	8943 (87.3)
<18.5 kg·m^−2^	331 (5.4)	766 (8.6)
≥18.5–<25 kg·m^−2^	2045 (33.1)	3596 (40.2)
≥25–<30 kg·m^−2^	1948 (31.5)	2495 (27.9)
≥30 kg·m^−2^	1855 (30.0)	2086 (23.3)
**Active asthma^¶^**	866 (13.5)	486 (4.7)
**Active rhinitis^¶^**	114 (1.8)	493 (4.8)
**Active eczema^¶^**	267 (4.2)	430 (4.2)
**Nasal polyps^+^**	133 (2.1)	147 (1.4)
**Diabetes mellitus^+^**	1132 (17.6)	1881 (18.4)
**GORD^+^**	1426 (22.2)	2044 (20.0)
**Cardiovascular disease^+^**	3001 (46.8)	6414 (62.6)
**Hypertension^+^**	3019 (47.1)	4886 (47.7)
**Osteoporosis^+^**	695 (10.8)	1241 (12.1)
**Depression/anxiety^+^**	2713 (42.3)	4396 (42.9)
**FEV_1_ % pred**	53.9±20.1	50.9±20.9
Subjects	5873	7883
≥80% pred	637 (10.8)	771 (9.8)
≥50%–<80% pred	2734 (46.6)	2901 (36.8)
≥30%–<50% pred	1776 (30.2)	3046 (38.6)
<30% pred	726 (12.4)	1165 (14.8)
**GOLD group**		
Subjects	5460	8789
A	838 (15.3)	556 (6.3)
B	768 (14.1)	701 (8.0)
C	1827 (33.5)	3641 (41.4)
D	2027 (37.1)	3891 (44.3)

Data are presented as n, mean±sd or n (%), unless otherwise stated. BMI: body mass index; GORD: gastro-oesophageal reflux disease; FEV_1_: forced expiratory volume in 1 s; GOLD: Global Initiative for Chronic Obstructive Lung Disease. ^#^: the closest BMI within 10 years of the index date, and the smoking status closest to and within 5 years of index date were included; ^¶^: active disease was defined as a diagnostic or monitoring read code or evidence for specific treatment within 1 year before index date; ^+^: ever diagnosed.

**FIGURE 1:**
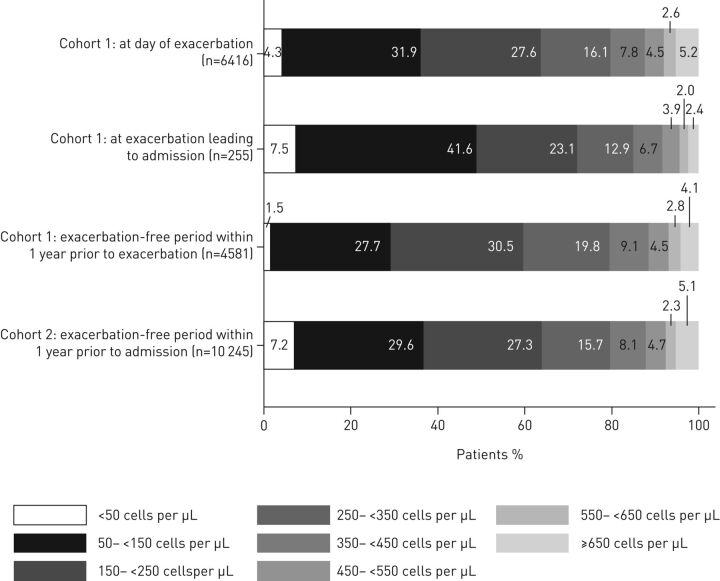
Distribution of blood eosinophil counts for Cohorts 1 and 2.

In patients treated with OCS for COPD exacerbations in a primary-care setting (Cohort 1), 1.5% had eosinopenia (low BEC <50 cells·µL^−1^) during an exacerbation-free period in the 12 months prior to the index date (figure 1). In comparing eosinophil distributions, percentages of patients who had BEC that were low (<50 cells·µL^−1^ [eosinopenia] and 50–<150 cells·µL^−1^) or very high (≥650 cells·µL^−1^) were greater on the day of an exacerbation than during an exacerbation-free period. Of the patients treated with OCS for COPD exacerbations in a primary-care setting (Cohort 1) who were hospitalised as a result of the index OCS-treated COPD exacerbation (n=255), almost half had BEC <150 cells·µL^−1^ at the time of their exacerbations (index date). For patients with HES linkage, incidence of overall treatment failure within 6 weeks was 44.4% (1103/2482), and incidence of hospitalisation for COPD was 10.3% (255/2482; table 2). For all patients treated with OCS for COPD exacerbations in a primary-care setting (Cohort 1), repeat OCS prescriptions were observed for 34.1% of patients (2187/6416) and antibiotic prescriptions for 9.7% (623/6416) during the 6 weeks after the index date. Antibiotic prescriptions were provided to 11.5% of patients who had been treated initially with OCS plus antibiotics and 6.9% (172/2499) who had not. Patients treated with ICS were at significantly greater risk of an additional antibiotics course within 6 weeks of exacerbation compared with patients not treated with ICS (10.4% *versus* 7.3%, p=0.001; table 2). ICS use was not associated with significant differences in any other outcomes.

**TABLE 2:** Treatment failure overall and for patients with or without inhaled corticosteroid (ICS) prescriptions in the baseline year for patients treated with oral corticosteroids (OCS) for COPD exacerbations in a primary-care setting (Cohort 1) and patients hospitalised due to COPD exacerbations (Cohort 2)

**Outcome**	**Population size n**	**Failure overall**	**Failure with ICS**	**Failure without ICS**	**p-value ICS *versus* no ICS**
**Cohort 1 (n=2482)**					
Overall treatment failure^#^	2482^¶^	1103 (44.4)	914 (45.0)	189 (42.0)	0.250
Hospital admission for COPD	2482^¶^	255 (10.3)	218 (10.7)	37 (8.2)	0.113
OCS repeat prescription	6416	2187 (34.1)	1752 (34.6)	435 (32.3)	0.118
Antibiotic prescription	6416	623 (9.7)	525 (10.4)	98 (7.3)	0.001
**Cohort 2 (n=10 245)**					
Hospital readmission	10 245	1189 (11.6)	986 (11.9)	203 (10.5)	0.084

Data are presented as n (%) unless otherwise stated. HES: Hospital Episode Statistics. ^#^: overall treatment failure for patients with HES linkage; treatment failure was defined as a COPD-related hospital admission or accident and emergency department visit, prescription for antibiotics with lower respiratory consultation or a repeat prescription for OCS. ^¶^: patients with HES linkage.

#### Association between BEC and oral corticosteroid-treated COPD exacerbation and treatment failure

**FIGURE 2:**
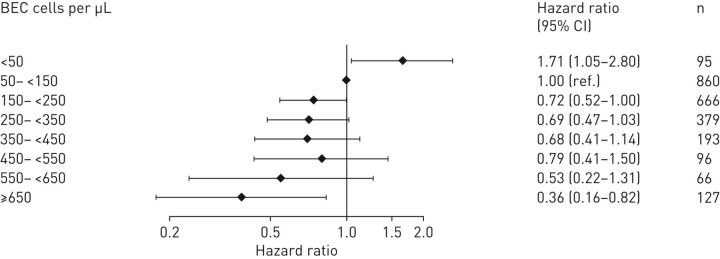
Adjusted hazard ratio for hospital admission within 6 weeks by blood eosinophil counts on the day of exacerbation for patients treated with oral corticosteroids for COPD exacerbations in a primary-care setting (Cohort 1). Patients categorised to eight eosinophil count categories as compared with a reference category of blood eosinophil counts 50 cells·µL^−1^–<150 cells·µL^−1^. BEC: blood eosinophil counts.

For patients treated with OCS for COPD exacerbations in a primary-care setting (Cohort 1), an association was observed between BEC on the day of an OCS-treated COPD exacerbation in primary care and the risk of subsequent hospitalisation for COPD exacerbation (figure 2). Patients with eosinopenia had the greatest risk of hospitalisation, with 19/95 (20.0%) being admitted to the hospital within 6 weeks (HR=1.71; 95% CI: 1.05–2.80, p=0.034, compared with the reference category 50–<150 cells·µL^−1^; figure 2). Kaplan–Meier estimates for hospital admissions or treatment failure within 6 weeks of the index date suggest that patients with eosinopenia were hospitalised or failed treatment earlier than others (supplementary figure S2). Of these, 42.1% (8/19) were hospitalised within 3 days. Patients with BEC ≥650 cells·µL^−1^ had the least risk of hospitalisation within 6 weeks (HR=0.36; 95% CI: 0.16–0.82). BEC increase of 100 cells·µL^−1^ was associated with a reduced risk of hospital admission (HR=0.26; 95% CI: 0.12–0.56, per 100-cells·µL^−1^ increase) when analysed as a continuous variable (table 3).

**TABLE 3:** Predictors of treatment failure (hospital admission for patients treated with oral corticosteroids for COPD exacerbation in a primary-care setting (Cohort 1), hospital readmission for patients hospitalised due to COPD exacerbations (Cohort 2))

**Predictor**	**Cohort 1**		**Cohort 2**	
	**HR (95% CI)**	**p-value**	**HR (95% CI)**	**p-value**
**Subjects n**	2482		10 245	
**Blood eosinophil count per 100-cells·µL^−1^ increase**	0.26 (0.12–0.56)	<0.001	0.78 (0.63–0.96)	0.020
**Male**	1.53 (1.19–1.98)	0.001	1.15 (1.03–1.29)	0.016
**Age per 10 years**			1.24 (1.17–1.32)	<0.001
**Underweight, BMI <18.5 kg·m^−2^**	2.29 (1.53–3.43)	<0.001	1.41 (1.17–1.70)	<0.001
**Smoking habits**				
Current smoker	1.74 (1.04–2.91)	0.034		
Ex-smoker	1.66 (1.02–2.71)	0.042		
**Cardiovascular disease**	2.18 (1.61–2.95)	<0.001		
**COPD exacerbations per 1 increase**	1.12 (1.07–1.17)	<0.001	1.09 (1.06–1.12)	<0.001
**mMRC dyspnoea scale score**				
Maximum score	1.69 (1.05–2.70)	0.030	1.28 (1.06–1.55)	0.012
Missing^#^			1.25 (1.06–1.48)	0.008
**FEV_1_ % pred**				
<30%			1.36 (1.15–1.62)	<0.001
Missing^#^			1.26 (1.09–1.45)	0.008
**Triple therapy^¶^** **prescribed**			1.19 (1.05–1.34)	0.005

HR: hazard ratio; BMI: body mass index; mMRC: modified Medical Research Council; FEV_1_: forced expiratory volume in 1 s.^#^: patients without monitoring data; ^¶^: triple therapy consisted of inhaled corticosteroids, long-acting β_2_-agonists and long-acting muscarinic antagonists.

Hazard ratios experiencing treatment failure for patients in each eosinophil count category are presented in supplementary figure S3. No relationship was found between BEC and additional OCS (HR=0.88; 95% CI: 0.73–1.07, p=0.204) or antibiotic prescriptions (HR=1.25; 95% CI: 0.91–1.73, p=0.168). However, patients with BEC ≥150 cells·µL^−1^ had an 11% lesser risk of repeat OCS prescriptions (HR=0.89; 95% CI: 0.81–0.97, p=0.010) and a 22% greater risk of additional antibiotics than patients with BEC 50–<150 cells·µL^−1^ (HR=1.22; 95% CI: 1.04–1.44, p=0.015). Risk of overall treatment failure was greatest for patients with eosinopenia (HR=1.58; 95% CI: 1.20–2.07, p=0.001) and least for patients with BEC ≥150 cells·µL^−1^ (HR=0.86; 95% CI: 0.76–0.98, p=0.026) compared with reference category 50–<150 cells·µL^−1^. The association between BEC and hospitalisation risk was significant for patients who were not treated with antibiotics (HR=0.12; 95% CI: 0.03–0.57, p=0.008), as well as for patients who were treated with antibiotics (HR=0.32; 95% CI: 0.13–0.78, p=0.012). We found no evidence that the association was modified by antibiotic treatment (p=0.465 for interaction term of antibiotic treatment and BEC in full model). The association between BEC and hospitalisation risk was also significant for patients without active asthma treated in a primary-care setting (Cohort 1) (HR=0.26; 95% CI: 0.11–0.59, p=0.001).

**FIGURE 3:**
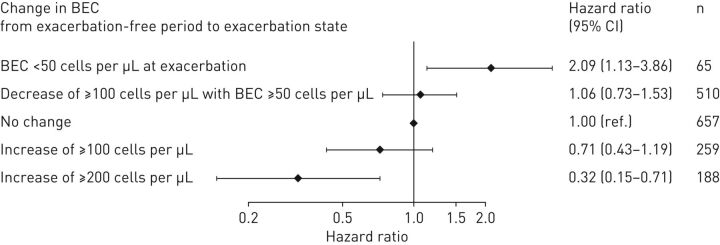
Association between hospital admission within 6 weeks and individual change in blood eosinophil counts from an exacerbation-free period to the day of exacerbation for patients treated with oral corticosteroids for COPD exacerbations in a primary-care setting (Cohort 1). BEC: blood eosinophil counts.

Of patients treated with OCS for COPD exacerbations in a primary-care setting (Cohort 1) who had data for BEC during an exacerbation-free period in the prior year and at index exacerbation, those with BEC <50 cells·µL^−1^ on the day of an exacerbation had the greatest risks of overall treatment failure and hospitalisation (figure 3, supplementary figure S4). Patients with increases ≥200 cells·µL^−1^ during exacerbations had relatively low risk of hospitalisation (HR=0.32; 95% CI: 0.15–0.71, p=0.006; figure 3).

A sensitivity analysis by ICS treatment in baseline year was performed for patients treated with OCS for COPD exacerbations in a primary-care setting (Cohort 1) (supplementary figures S5 and S6). Effects of BEC on risk of hospital admission or treatment failure by any definition were similar for patients who did and did not receive ICS in the prior year.

### Patients hospitalised due to COPD exacerbations (Cohort 2)

In patients hospitalised due to COPD exacerbations (Cohort 2), index years ranged from 1997 to 2016. We identified 11 405 patients who were hospitalised for COPD exacerbations and had BEC recorded during an exacerbation-free period in the prior year (see supplementary figure S7 for patient flow diagram). A total of 1160 patients (10.2%) died in the hospital during the index admission for COPD, leaving 10 245 patients who were eligible for inclusion. Most patients in Cohort 2 were aged from 60 to <80 years (table 1).

In patients hospitalised for COPD exacerbation (Cohort 2), 7.2% of patients had eosinopenia (BEC <50 cells·µL^−1^) during an exacerbation-free period in 12 months prior to the index date (figure 1). Of hospitalised patients (Cohort 2), 11.6% (1189/10 245) were readmitted to the hospital within 4 weeks for COPD exacerbation (table 2). Frequency of hospital readmission was not substantially different between patients who did and did not receive ICS treatment in the previous year.

#### Association between BEC during an exacerbation-free period in the year prior to index date and hospital readmission in patients hospitalised for COPD exacerbations (Cohort 2)

**FIGURE 4:**
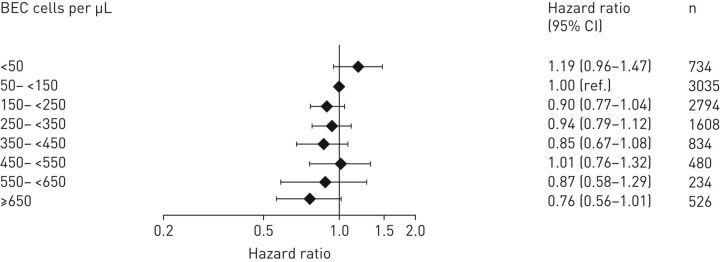
Adjusted hazard ratio for hospital readmission within 4 weeks by blood eosinophil counts during an exacerbation-free period in the prior year for patients hospitalised due to COPD exacerbations (Cohort 2). BEC: blood eosinophil counts.

Kaplan–Meier estimates for hospital admissions suggest that patients with eosinopenia were hospitalised earlier than patients with greater BEC (supplementary figure S8). Risk of short-term hospital readmission for COPD exacerbation within 4 weeks was greatest for patients with BEC <50 cells·µL^−1^ (14.7% (108/734); HR=1.19; 95% CI: 0.96–1.47, p=0.119) and least for patients with BEC ≥650 cells·µL^−1^ (9.7% (51/526); HR=0.76; 95% CI: 0.56–1.01, p=0.063; figure 4). When analysed as a continuous variable, risk of short-term readmission decreased significantly with increasing BEC (HR=0.78; 95% CI: 0.63–0.96, p=0.020 per 100-cells·µL^−1^ increase (table 3). The association between risk and BEC was more pronounced for the 5449 (47.8%) patients with a history of OCS treatment in the prior year (HR=0.52; 95% CI: 0.33–0.80, p=0.003). ICS treatment had no apparent effect on risk of hospital readmission (supplementary figure S5). The association between BEC and hospital readmission risk remained significant after exclusion of patients with active asthma (HR=0.80; 95% CI: 0.65–0.99, p=0.039). No relevant differences in the use of maintenance therapy and ICS dosages between incremental categories of BECs at baseline were found (supplementary table S2).

**FIGURE 5:**
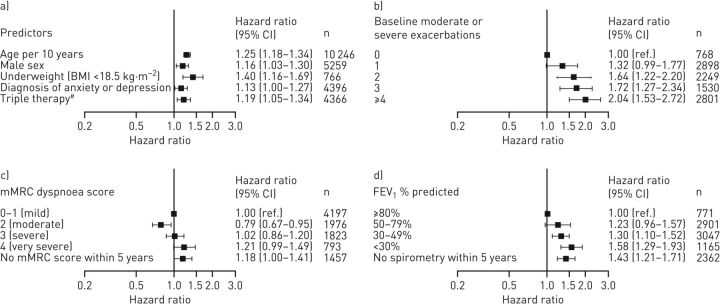
Adjusted hazard ratio for hospital readmission within 4 weeks by baseline characteristics for patients hospitalised due to COPD exacerbations (Cohort 2). a) Predictors; b) baseline moderate or severe exacerbations; c) modified Medical Research Council (mMRC) dyspnoea score; d) forced expiratory volume in 1s (FEV_1_) % predicted. BMI: body mass index. ^#^: triple therapy consisted of inhaled corticosteroids, long-acting β_2_-agonists and long-acting muscarinic antagonists.

Risk of hospital readmission increased 25% with every 10-year increase in patient age (figure 5). This risk was also greater for patients who were male, underweight (BMI <18.5), receiving triple therapy, and who had a diagnosis of anxiety or depression. Patients who experienced ≥4 exacerbations during the year prior to the index date had twice the risk of hospital readmission compared with patients who experienced no exacerbations in that time. Severe dyspnoea was associated with a greater hazard ratio for hospital readmission. This risk also increased as FEV_1_ decreased (figure 5).

## Discussion

Overall, the data from the two cohorts demonstrated that patients with greater BEC had better treatment response and outcomes to OCS than those with eosinopenia. For patients treated with OCS for COPD exacerbations in a primary-care setting (Cohort 1), steroids were most effective in preventing hospital admission for patients with BEC increases ≥200 cells·µL^−1^ from an exacerbation-free period to exacerbation state. Patients with greater BEC on the day of an exacerbation who were treated with OCS in primary care had a lesser risk of hospital admission. For patients hospitalised due to COPD exacerbations (Cohort 2), our data suggest that patients with BEC ≥650 cells·µL^−1^ during an exacerbation-free period were most responsive to OCS treatment upon hospital admission for exacerbation. Our study supports the findings of Bafadhel *et al*. [10] that OCS treatment for exacerbations does not have a clear benefit for patients with eosinopenia and supports the positive results of eosinophil-guided corticosteroid therapy in the CORTICO-COP trial [11]. For patients with eosinopenia who do not benefit from OCS, exacerbations are not likely to be driven by eosinophils and may require a different treatment approach [23].

In contrast, eosinopenia was associated with poorer patient outcomes, including hospital admission within a few days of COPD exacerbation and short-term readmission after discharge. The risk of both outcomes decreased with increasing BEC in a count-dependent manner. The risk of overall treatment failure was also greatest for patients with eosinopenia. Patients whose BEC decreased to <150 cells·µL^−1^ also had elevated risk of hospitalisation for exacerbations. A decrease in BEC, which can occur with acute bacterial infection and inflammation, is considered a reliable clinical marker of acute infection [24]. Eosinopenia not caused by prior OCS treatment has been recognised as a marker of sepsis and may indicate that patients are critically ill and require immediate antibiotic treatment [25]. Identification of the different drivers of the exacerbation (*e.g.*, bacterial infection), together with biomarkers and clinical features, can support treatment choice and improve treatment outcomes.

Our findings concerning blood eosinophils agree with several published studies. Duman *et al*. [17] found that patients with elevated blood eosinophils (≥2%) had shorter hospital stays and lesser readmission rates over 6 months. Bafadhel *et al*. [7] concur that patients who were hospitalised for COPD exacerbations with BEC ≥200 cells·µL^−1^ had shorter stays than patients with eosinopenia. However, this did not impact long-term health outcomes or readmission rates. A recent study by Prins *et al*. [16] confirmed the greater treatment response by demonstrating reduced risk of treatment failure within 10 days for patients with blood eosinophils ≥2% on admittance (with a sensitivity analysis with ≥300 cells·µL^−1^), but found a significantly increased risk of relapse after >30 days of follow-up. However, other studies describe increased risks of hospital readmission or treatment failure for patients with elevated BEC on admission [15, 16]. The different data sources, inclusion criteria and analyses used for these studies may account for some of the differences in findings and do not allow for direct comparisons between studies. Prospective studies are required to provide more insight into the effects of BEC on treatment outcomes for patients with COPD. Our findings that some patients with relatively elevated BEC (≥450 cells·µL^−1^) and a recent history of OCS use were hospitalised and appeared to have responded well to more intensive OCS treatment during hospitalisation are consistent with previous findings. Evidence suggests that patients with BEC ≥450 cells·µL^−1^ had increased exacerbation rates [26] and required more intensive treatment than routine care.

Our studies used real-world data from validated, high-quality databases, in addition to primary-care data enhanced by the influence of the UK Quality and Outcomes Framework [27] and detailed diagnostic reasons for hospital admission from HES [28]. Inclusion criteria were selected for patients with managed COPD in advance of admission rather than COPD diagnosed at the time of admission. However, uncertainty remains as to whether these patients with BEC recorded on the day of an exacerbation are representative of all patients with exacerbations, as BEC testing is not routine. The primary outcome for patients treated with OCS for COPD exacerbations in the primary-care setting (Cohort 1) includes hospital admissions as a valid proxy for treatment failure. Additional prescriptions are a less certain measure of treatment failure because general practitioners may prescribe another OCS treatment course for patients with persistent symptoms but not initial treatment failure. Another study limitation was the lack of patient information on spirometry and smoking habits, which may have led to the inclusion of patients with asthma. Although treatment of exacerbations with OCS is a well-established, common practice to reduce the risk of treatment failure or prolonged hospital stay during hospitalisation for patients with COPD [29, 30], an additional study limitation was the lack of information on treatment received during hospitalisation. Additionally, the lack of treatment information after hospital discharge and before readmission renders the association between hospital readmission and BEC values obtained during an exacerbation-free period in the year prior to index hospitalisation as a hypothesis-generating path. Finally, statistical power was limited in these analyses, particularly when evaluating short-term readmission rates, and larger prospective studies may be required to confirm our findings.

Standard management of COPD exacerbations with OCS has limited success for some patients. COPD exacerbations have been associated with bacteria, viruses and sputum eosinophilia [5]. The response to OCS therapy as part of hospitalisation treatment or community treatment is predicted by eosinophil counts. Patients with the lowest eosinophil counts likely do less well, and those with greater eosinophil counts probably have a greater treatable trait and thus may respond to OCS. To some extent, this response is consistent with the data on ICS as maintenance therapy. Low eosinophil counts may be a marker of acute or chronic bacterial infection and may predict the necessity of antibiotic therapy. There are published data on this for hospitalised patients, but little for community-treated COPD exacerbations. We found that BEC may be biomarkers that can aid in treatment decisions for patients with COPD with greater BEC indicating responsiveness to OCS to treat acute exacerbations. In addition, BEC <150 cells·µL^−1^ may indicate an underlying condition or set of conditions associated with treatment failure and worse outcomes. Together, these data suggest that current practices of OCS treatment for COPD exacerbations should be reconsidered. Biomarkers together with clinical features can support physicians’ treatment decisions.

## Supplementary material

10.1183/23120541.00188-2020.Supp1 **Please note:** supplementary material is not edited by the Editorial Office, and is uploaded as it has been supplied by the author.
Supplementary material 00188-2020.SUPPLEMENT
