# Supplementary material for: Blood eosinophil count predicts treatment failure and hospital readmission for COPD
Source: ERJ Open Res. 2020 Nov 10;6(4):00188-2020. doi: 10.1183/23120541.00188-2020 (PMC7927786; doi:10.1183/23120541.00188-2020)
Supplement: Supplementary file 1 [file 00188-2020.SUPPLEMENT.pdf]

Blood Eosinophil Count Predicts Treatment Failure and Hospital Readmission for COPD

Marjan Kerkhof[1], Isha Chaudhry[1], Ian D. Pavord[2], Marc Miravitlles[3], Chin Kook Rhee[4], David M.G. Halpin[5], Omar S. Usmani[6], Rupert Jones[7], Janwillem Kocks[1], Marianna Alacqua[8], Tamsin Morris[9], Alan Kaplan[1,10], David B. Price[1,11]

[1]Observational and Pragmatic Research Institute, Singapore; [2]Oxford Respiratory NIHR BRC, Nuffield Department of Medicine, University of Oxford, Oxford, United Kingdom; [3]Pneumology Department, Hospital Universitari Vall d'Hebron, CIBER de Enfermedades Respiratorias (CIBERES), Barcelona, Spain; [4]College of Medicine, Seoul St. Mary's Hospital, The Catholic University of Korea, Seoul, South Korea; [5]University of Exeter Medical School, College of Medicine and Health, University of Exeter, Exeter, United Kingdom; [6]Imperial College London, London, United Kingdom; [7]The Peninsula College of Medicine and Dentistry, Plymouth, United Kingdom; [8]AstraZeneca, Cambridge, United Kingdom; [9]AstraZeneca, Luton, United Kingdom; [10]Family Physician Airways Group of Canada, Richmond Hill, Ontario, Canada; [11]University of Aberdeen, Aberdeen, United Kingdom

Table of Contents

Table S1. Additional Baseline Patient Characteristics 3

Table S2. Baseline Patient Maintenance Therapy by Blood Eosinophil Count Category 5

Figure S1. Cohort 1 Patient Flow Diagram 6

Figure S2. Kaplan-Meier Curves for the Unadjusted Association Between Blood Eosinophil Counts Recorded on the Day of an OCS-Treated Exacerbation and Time to First Outcome Event Within 6 Weeks (Cohort 1) 8

Figure S3. Association Between Blood Eosinophil Counts Recorded on the Day of an OCS-Treated Exacerbation and Treatment Failure Within 6 Weeks (Cohort 1) 9

Figure S4. Association Between Change in Blood Eosinophil Counts from an Exacerbation-Free Period to Index Exacerbation Date and Treatment Failure Within 6 Weeks (Cohort 1) 11

Figure S5. Association Between Blood Eosinophil Counts and Hospital Admission Within 6 Weeks (Cohort 1) or Readmission Within 4 Weeks (Cohort 2) Stratified by ICS Treatment in the Prior Year 13

Figure S6. Association Between Blood Eosinophil Counts and Treatment Failure Outcomes Within 6 Weeks, Stratified by ICS Treatment (Cohort 1) 14

Figure S7. Cohort 2 Patient Flow Diagram 16

Figure S8. Kaplan-Meier Curves for the Unadjusted Association Between Blood Eosinophil Counts Recorded During an Exacerbation-Free Period and Time to First Hospital Readmission for COPD Exacerbation (Cohort 2) 17

Table S1. Additional Baseline Patient Characteristics

Variable	Cohort 1 (N=6,416)	Cohort 2 (N=10,245)
Charlson comorbidity index		
≤1	4,023 (62.7)	6,199 (60.5)
2–4	1,344 (20.9)	1,730 (16.9)
5+	1,049 (16.3)	2,316 (22.6)
Maintenance therapy		
None	755 (11.8)	1,253 (12.2)
Short-acting BD only	592 (9.2)	687 (6.7)
Long-acting BD only	680 (10.6)	1,232 (12.0)
ICS+LABA	1,500 (23.4)	2,674 (26.1)
Triple therapy	2,889 (45.0)	4,399 (42.9)
Daily ICS dosage, median (IQR)[a]	361.7 (82.0–721.3)	393.5 (92.9–739.8)
≥1 SABA inhaler prescribed	4,687 (73.1)	7,594 (74.1)
≥1 SAMA inhaler prescribed	1,066 (16.6)	3,563 (34.8)
Cumulative average daily OCS dosage, median (IQR)[b]	1.5 (0.8–2.9)	0.4 (0.0–1.4)
Number of COPD exacerbations[c]		
0	0 (0)	0 (0)
1	520 (8.1)	2,348 (22.9)
2	2,331 (36.3)	2,325 (22.7)
3	1,155 (18.0)	1,831 (17.9)
≥4	2,410 (37.6)	3,741 (36.5)
Number of short-term OCS courses[c]		
0	0 (0)	4,796 (46.8)
1	1,946 (30.3)	2,332 (22.8)
2	1,225 (19.1)	1,215 (11.9)
3	945 (14.7)	753 (7.3)
≥4	2,300 (35.8)	1,149 (11.2)
mMRC dyspnoea score		
n	5,460	8,789
0	409 (7.5)	314 (3.6)
1	2,256 (41.3)	3,883 (44.2)
2	1,527 (28.0)	1,976 (22.5)
3	1,030 (18.9)	1,823 (20.7)
4	238 (4.4)	793 (9.0)

BD, bronchodilator; COPD, chronic obstructive pulmonary disease; ICS, inhaled corticosteroids; IQR, interquartile range; LABA, long-acting β_2-agonists; mMRC, modified Medical Research Council; OCS, oral corticosteroids; SABA, short-acting β_2-agonists; SAMA, short-acting muscarinic antagonists.

Data expressed as n (%) unless otherwise noted.

[a]ICS dosage expressed as fluticasone propionate or equivalent (µg).

[b]OCS dosage expressed as prednisolone or equivalent (mg).

[c]Including exacerbation on index date.

Table S2. Baseline Patient Maintenance Therapy by Blood Eosinophil Count Category

		Blood eosinophil category (cells/µL)							
		**<50**	**50–<150**	**150–<250**	**250–<350**	**350–<450**	**450–<550**	**550–<650**	**≥650**
**Cohort 1**		**(n=275)**	**(n=2048)**	**(n=1768)**	**(n=1033)**	**(n=502)**	**(n=287)**	**(n=169)**	**(n=334)**
Maintenance therapy	None	37 (13.5)	262 (12.8)	209 (11.8)	107 (10.4)	54 (10.8)	33 (11.5)	15 (8.9)	38 (11.4)
	Short-acting BD only	30 (10.9)	193 (9.4)	145 (8.2)	106 (10.3)	45 (9.0)	30 (10.5)	16 (9.5)	27 (8.1)
	Long-acting BD only	35 (12.7)	220 (10.7)	180 (10.2)	97 (9.4)	46 (9.2)	33 (11.5)	27 (16.0)	42 (12.6)
	ICS+LABA	58 (21.1)	451 (22.0)	440 (24.9)	240 (23.2)	118 (23.5)	69 (24.0)	37 (21.9)	87 (26.0)
	Triple therapy	115 (41.8)	922 (45.0)	794 (44.9)	483 (46.8)	239 (47.6)	122 (42.5)	74 (43.8)	140 (41.9)
ICS mean daily dosage	Median (IQR)	731.5 (114.8–1530.1)	786.9 (164.4–1528.8)	794.0 (246.6–1506.8)	746.9 (197.0–1479.5)	788.0 (163.9–1479.5)	731.5 (164.4–1315.1)	657.5 (164.4–1315.1)	739.7 (164.4–1475.4)
**Cohort 2**		**(n=734)**	**(n=3035)**	**(n=2794)**	**(n=1608)**	**(n=834)**	**(n=480)**	**(n=234)**	**(n=526)**
Maintenance therapy	None	108 (14.7)	399 (13.1)	314 (11.2)	196 (12.2)	93 (11.2)	53 (11.0)	24 (10.3)	66 (12.5)
	Short-acting BD only	53 (7.2)	200 (6.6)	166 (5.9)	103 (6.4)	79 (9.5)	30 (6.3)	21 (9.0)	35 (6.7)
	Long-acting BD only	83 (11.3)	368 (12.1)	363 (13.0)	179 (11.1)	85 (10.2)	53 (11.0)	27 (11.5)	74 (14.1)
	ICS+LABA	200 (27.2)	778 (25.6)	696 (24.9)	447 (27.8)	209 (25.1)	126 (26.3)	61 (26.1)	157 (29.8)
	Triple therapy	290 (39.5)	1,290 (42.5)	1,255 (44.9)	683 (42.5)	368 (44.1)	218 (45.4)	101 (43.2)	194 (36.9)
ICS mean daily dosage	Median (IQR)	657.5 (54.8–1315.1)	723.3 (109.6–1479.5)	737.7 (164.4–1444.6)	710.4 (163.9–1315.1)	690.4 (164.4–1479.5)	657.5 (109.6–1315.1)	684.9 (137.0–1315.1)	657.5 (164.4–1479.5)

BD, bronchodilator; ICS, inhaled corticosteroids; IQR, interquartile range; LABA, long-acting β_2-agonists.

Figure S1. Cohort 1 Patient Flow Diagram

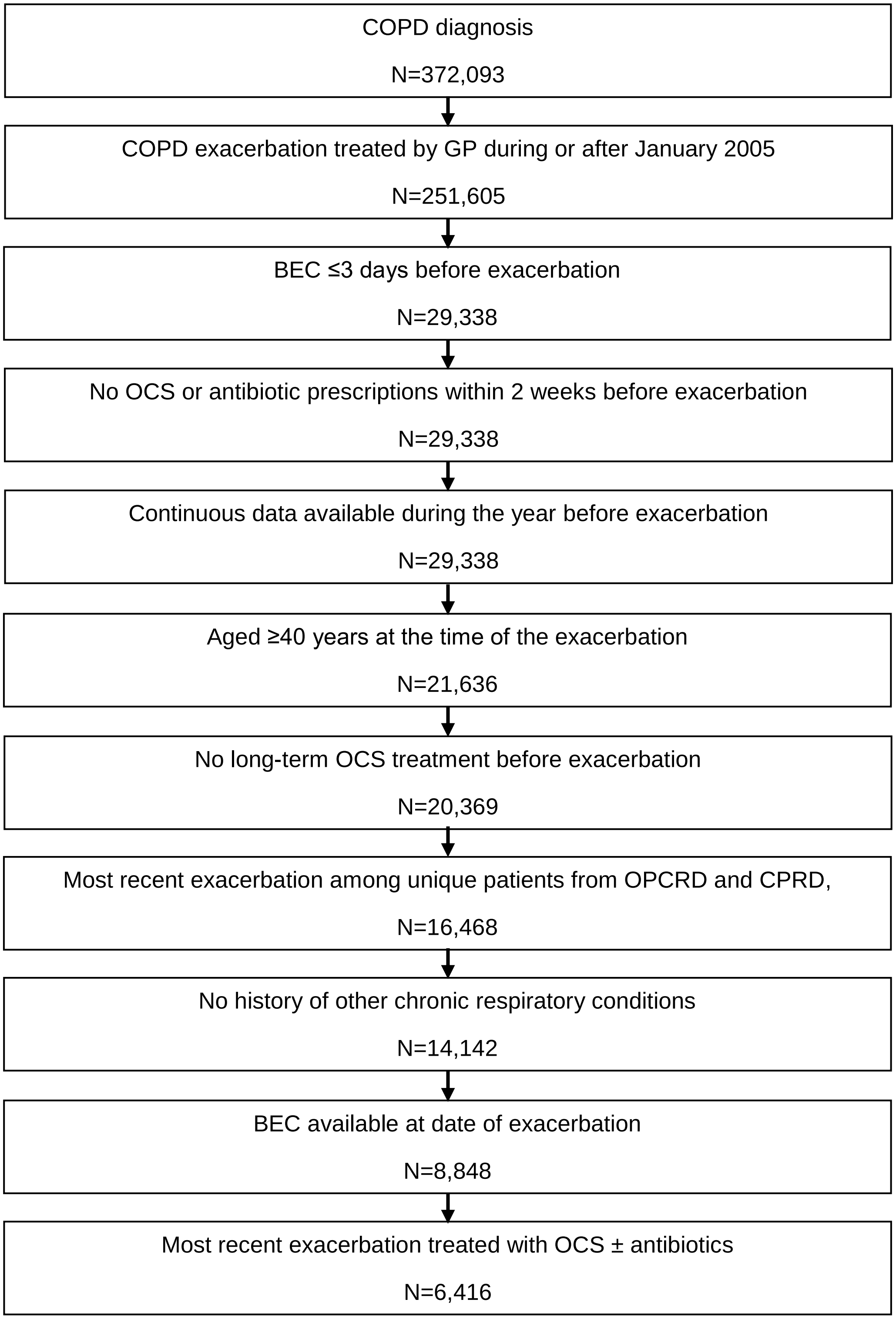

BEC, blood eosinophil counts; CPRD, Clinical Practice Research Datalink; COPD, chronic obstructive pulmonary disease; GP, general practitioner; OCS, oral corticosteroids; OPCRD, Optimum Patient Care Research Database.

Figure S2. Kaplan-Meier Curves for the Unadjusted Association Between Blood Eosinophil Counts Recorded on the Day of an OCS-Treated Exacerbation and Time to First Outcome Event Within 6 Weeks (Cohort 1)

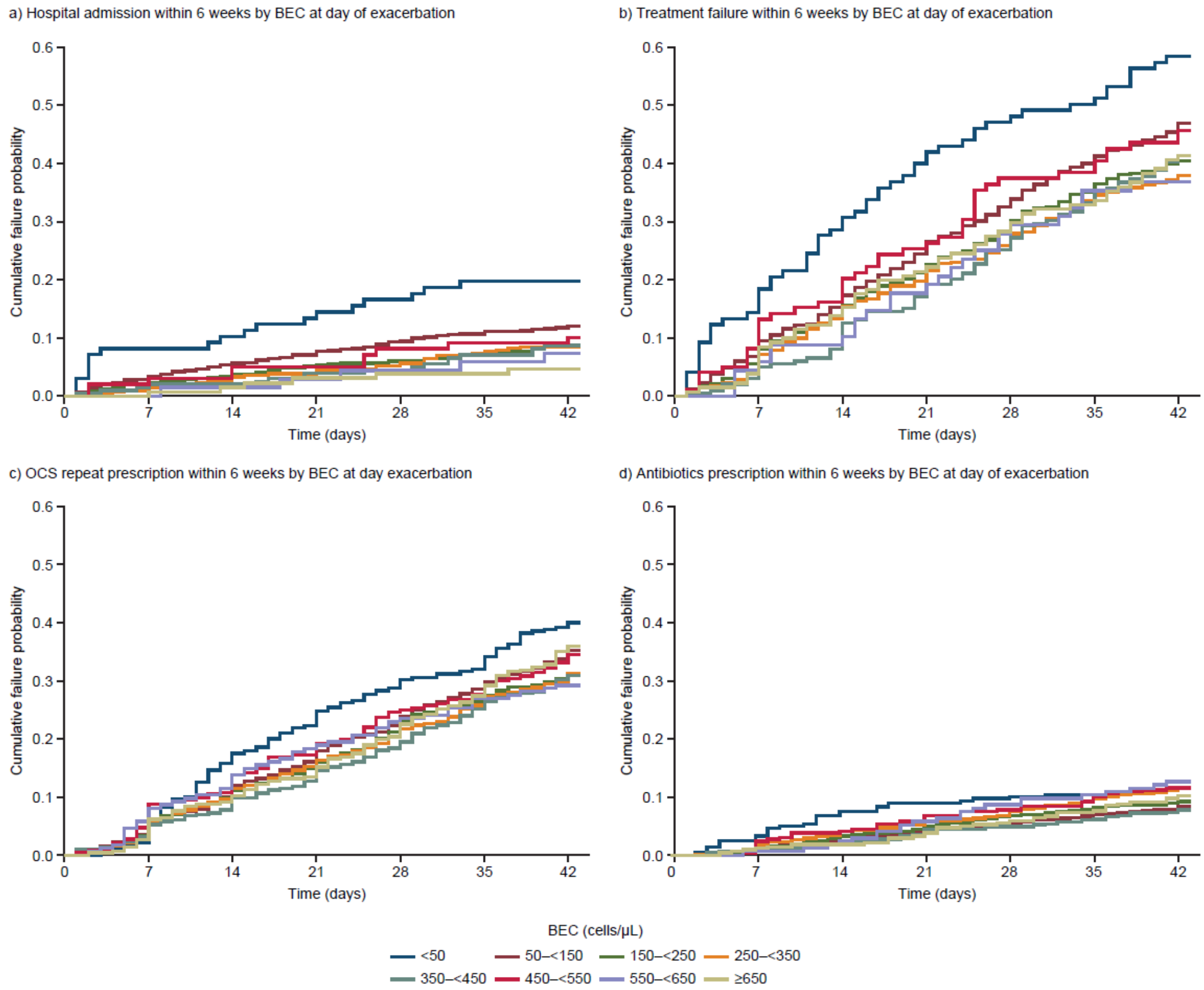

Panel a: Hospital admission. Panel b: Treatment failure. Panel c: OCS prescription.

Panel d: Antibiotic prescription.

BEC, blood eosinophil counts; OCS, oral corticosteroids.

BEC are classified in incremental categories with 50–<150 cells/μL as the reference category.

Figure S3. Association Between Blood Eosinophil Counts Recorded on the Day of an OCS-Treated Exacerbation and Treatment Failure Within 6 Weeks (Cohort 1)

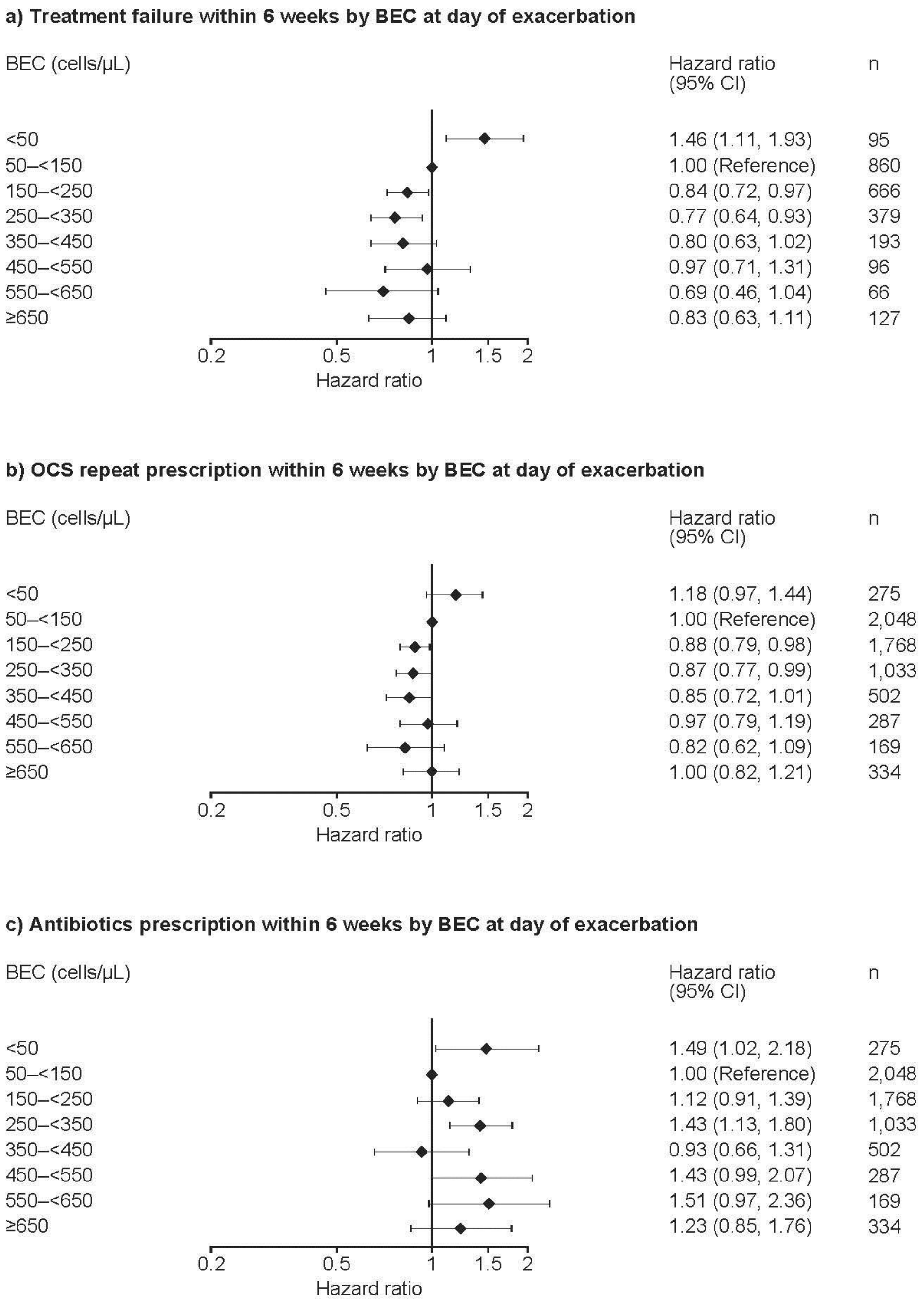

Panel a: Overall treatment failure (N=2,482). Panel b: Treatment failure defined as a repeat OCS prescription (N=6,416). Panel c: Treatment failure defined as an antibiotic prescription (N=6,416).

BEC, blood eosinophil counts; CI, confidence interval; OCS, oral corticosteroids.

Figure S4. Association Between Change in Blood Eosinophil Counts from an Exacerbation-Free Period to Index Exacerbation Date and Treatment Failure Within 6 Weeks (Cohort 1)

a) Treatment failure within 6 weeks by BEC at exacerbation vs. exacerbation-free period

Difference in BEC from exacerbation-free period to exacerbation	Hazard ratio (95% CI)	n
BEC <50 cells/µL at exacerbation	2.04 (1.45, 2.87)	65
Decrease of ≥100 cells/µL with BEC ≥50 cells/µL	1.24 (1.03, 1.48)	510
No change	1.00 (Reference)	657
Increase of ≥100 cells/µL	0.85 (0.66, 1.08)	259
Increase of ≥200 cells/µL	1.06 (0.82, 1.37)	188

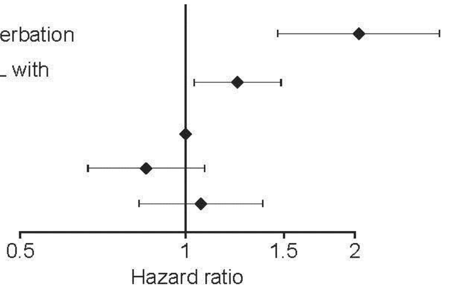

b) OCS repeat prescription within 6 weeks by BEC at exacerbation vs. exacerbation-free period

Difference in BEC from exacerbation-free period to exacerbation	Hazard ratio (95% CI)	n
BEC <50 cells/µL at exacerbation	1.20 (0.93, 1.54)	197
Decrease of ≥100 cells/µL with BEC ≥50 cells/µL	1.14 (1.00, 1.29)	1,381
No change	1.00 (Reference)	1,721
Increase of ≥100 cells/µL	0.83 (0.70, 0.97)	775
Increase of ≥200 cells/µL	1.06 (0.89, 1.27)	507

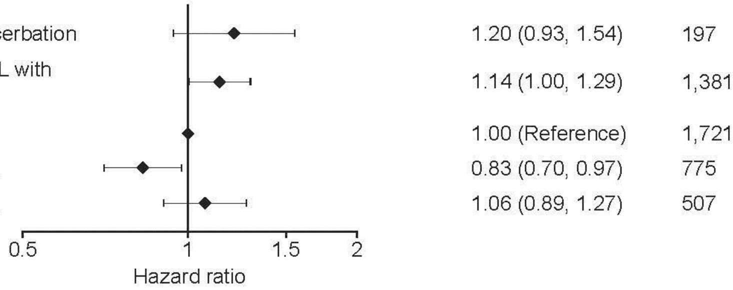

c) Antibiotics prescription within 6 weeks by BEC at exacerbation vs. exacerbation-free period

Difference in BEC from exacerbation-free period to exacerbation	Hazard ratio (95% CI)	n
BEC <50 cells/µL at exacerbation	1.27 (0.79, 2.05)	197
Decrease of ≥100 cells/µL with BEC ≥50 cells/µL	1.00 (0.78, 1.29)	1,381
No change	1.00 (Reference)	1,721
Increase of ≥100 cells/µL	1.01 (0.75, 1.36)	775
Increase of ≥200 cells/µL	1.24 (0.90, 1.71)	507

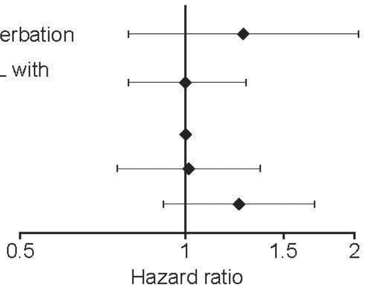

Panel a: Overall treatment failure. Panel b: Treatment failure defined as a repeat OCS prescription. Panel c: Treatment failure defined as an antibiotic prescription.

BEC, blood eosinophil counts; CI, confidence interval; OCS, oral corticosteroids.

An exacerbation-free period is defined as at least 4 weeks before any COPD exacerbations.

Figure S5. Association Between Blood Eosinophil Counts and Hospital Admission Within 6 Weeks (Cohort 1) or Readmission Within 4 Weeks (Cohort 2) Stratified by ICS Treatment in the Prior Year

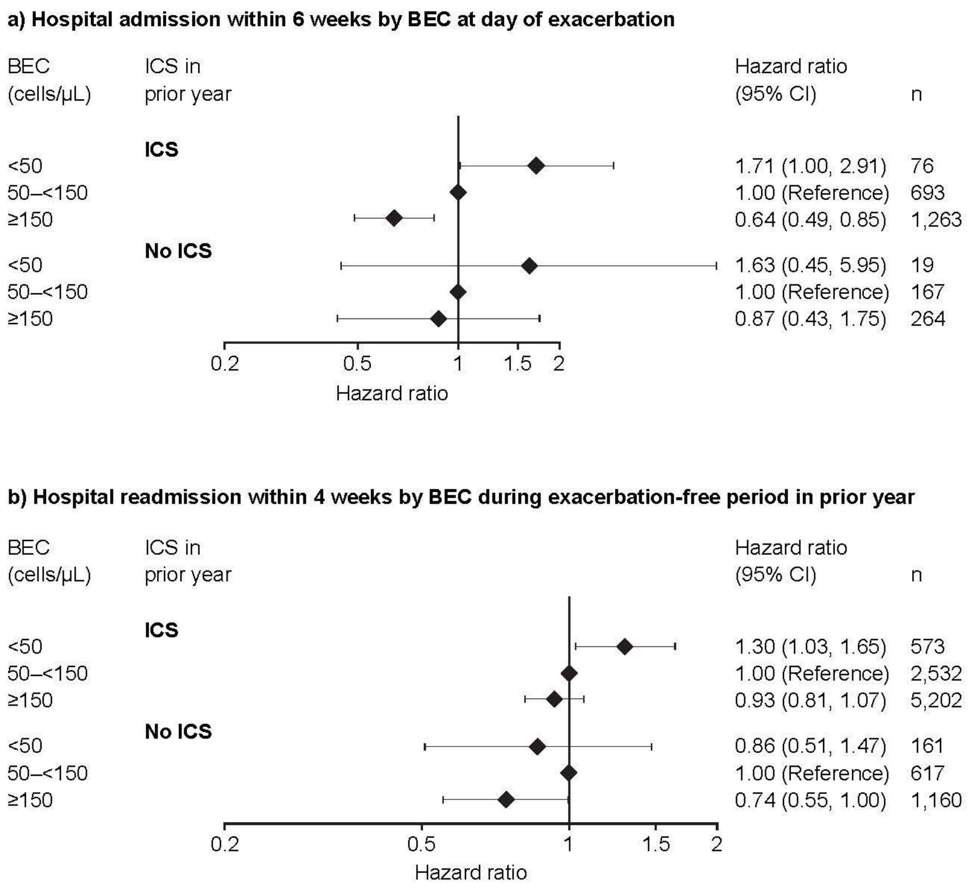

Panel a: Association between BEC recorded on the day of an exacerbation and hospital admission within 6 weeks for Cohort 1. Panel b: Association between BEC recorded during stable disease (no exacerbations within 4 weeks) and hospital readmission within 6 weeks for Cohort 2.

BEC, blood eosinophil counts; CI, confidence interval; ICS, inhaled corticosteroids.

Figure S6. Association Between Blood Eosinophil Counts and Treatment Failure Outcomes Within 6 Weeks, Stratified by ICS Treatment (Cohort 1)

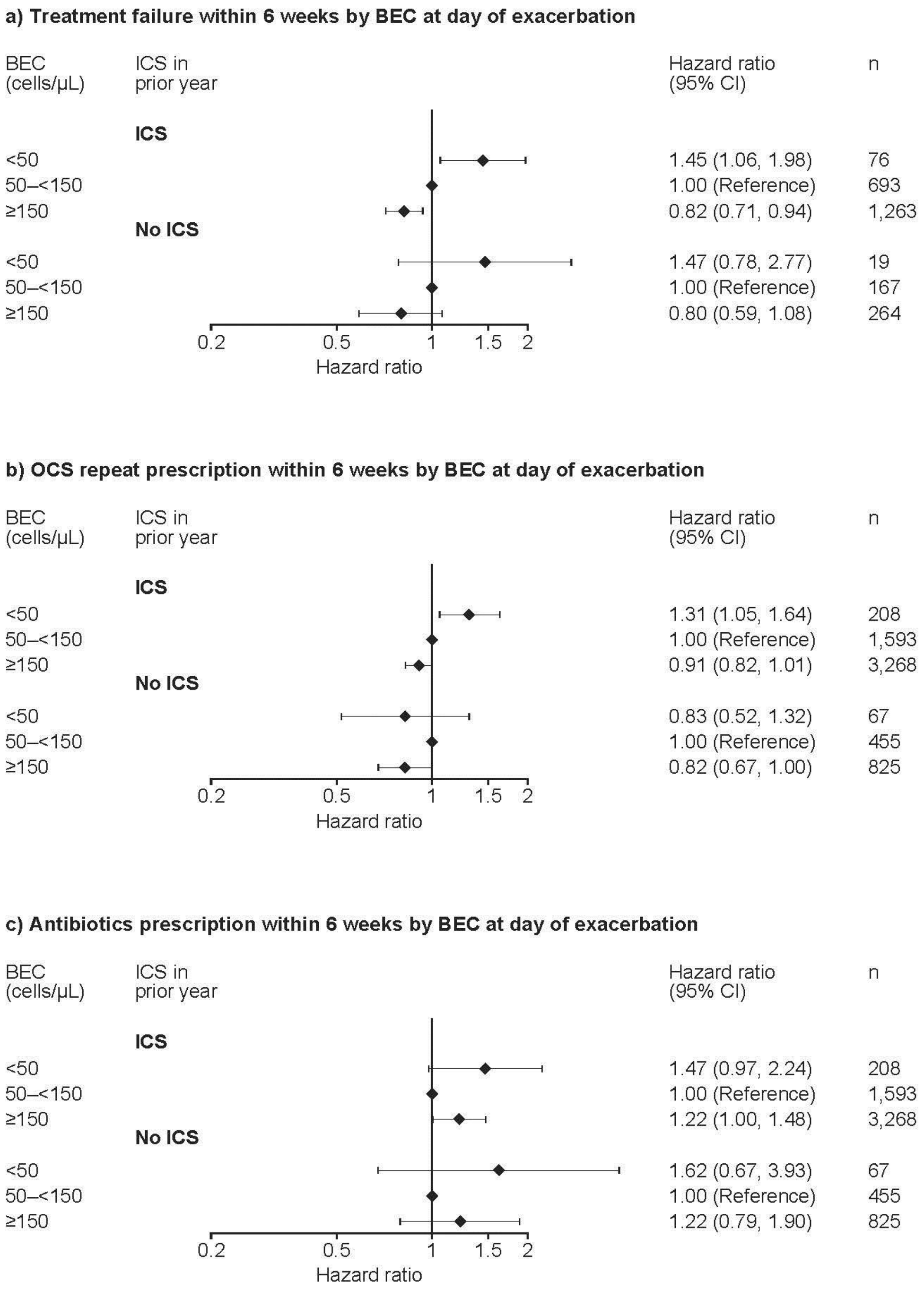

Panel a: Overall treatment failure. Panel b: Treatment failure defined as a repeat OCS prescription. Panel c: Treatment failure defined as an antibiotic prescription.

BEC, blood eosinophil counts; CI, confidence interval; ICS, inhaled corticosteroids; OCS, oral corticosteroids.

Reference range for BEC was 50–<150 cells/μL.

Figure S7. Cohort 2 Patient Flow Diagram

COPD diagnosis linked with HES data

N=128,290

≥1 hospital admission for COPD exacerbation within 1 year of BEC measurement

N=25,462

No COPD exacerbation within 4 weeks before BEC measurement

N=20,099

Registered at GP practice at hospital discharge date

N=19,086

Continuous data available during the year before hospital discharge date

N=18,887

Code of COPD/COPD monitoring review before hospital admission

N=13,340

Aged ≥40 years at hospital discharge date

N=13,333

No long-term OCS treatment before index date

N=12,080

No history of other chronic respiratory conditions

N=11,405

Patient discharged from hospital alive

N=10,245

BEC, blood eosinophil counts; COPD, chronic obstructive pulmonary disease; GP, general practitioner; HES, Hospital Episode Statistics; OCS, oral corticosteroids.

Figure S8. Kaplan-Meier Curves for the Unadjusted Association Between Blood Eosinophil Counts Recorded During an Exacerbation-Free Period and Time to First Hospital Readmission for COPD Exacerbation (Cohort 2)

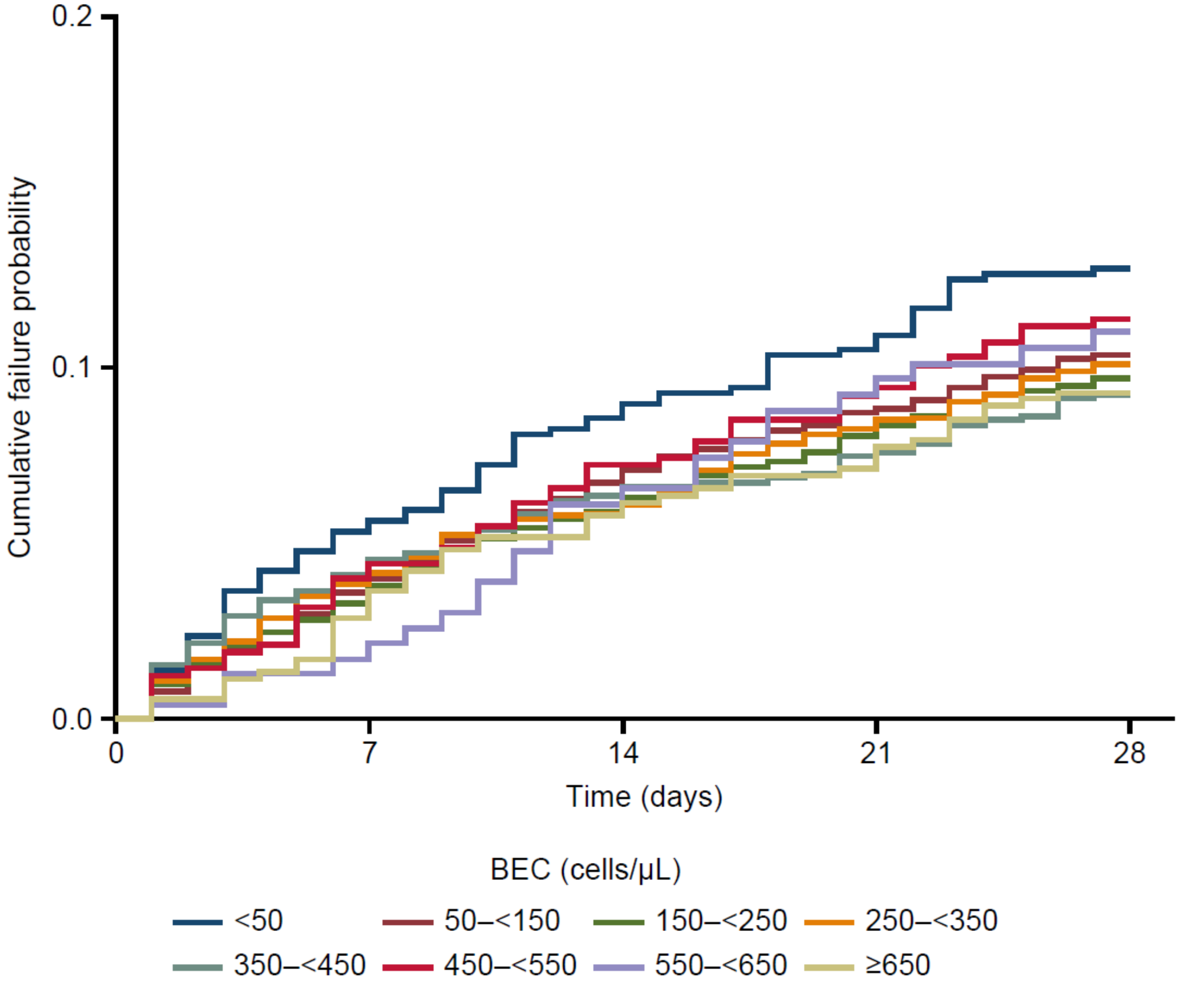

BEC, blood eosinophil counts; COPD, chronic obstructive pulmonary disease.

An exacerbation-free period was defined as at least 4 weeks before any COPD exacerbations.
